# Supplementary material for: Controlled Release of 18-β-Glycyrrhetinic Acid from Core-Shell Nanoparticles: Effects on Cytotoxicity and Intracellular Concentration in HepG2 Cell Line
Source: Materials (Basel). 2021 Jul 12;14(14):3893. doi: 10.3390/ma14143893 (PMC8303372; doi:10.3390/ma14143893)
Supplement: Supplementary file 1 [file materials-14-03893-s001.zip › materials-1276749-supplementary.pdf]

materials

MDPI

Supplementary materials

Controlled Release of 18-β-Glycyrrhetinic Acid from Core-Shell Nanoparticles: Effects on Cytotoxicity and Intracellular Concentration in HepG2 Cell Line

Giuseppina Nocca [1,2,*,†], Giuseppe D'Avenio [3,†], Adriana Amalfitano [1], Laura Chronopoulou [4,*], Alvaro Mordente [1,2], Cleofe Palocci [4,5,†] and Mauro Grigioni [3,†]

1 Dipartimento di Scienze biotecnologiche di base, Cliniche Intensivologiche e Perioperatorie, Università Cattolica del Sacro Cuore, 00168 Rome, Italy; adriana.amalfitano@unicatt.it (A.A.); alvaro.mordente@unicatt.it (A.M.)
2 Fondazione Policlinico Universitario A. Gemelli, IRCCS, 00168 Rome, Italy
3 National Center for Innovative Technologies in Public Health, Istituto Superiore di Sanità, 00161 Rome, Italy; giuseppe.davenio@iss.it (G.D.); mauro.grigioni@iss.it (M.G.)
4 Department of Chemistry, University La Sapienza, 00185 Rome, Italy; cleofe.palocci@uniroma1.it
5 CIABC-Centro di Ricerca per le Scienze Applicate alla Protezione dell'Ambiente e dei Beni Culturali, University La Sapienza, 00185 Rome, Italy
* Correspondence: giuseppina.nocca@unicatt.it (G.N.); laura.chronopoulou@uniroma1.it (L.C); Tel.: +39-06-3015-4215 (G.N.); Tel.:+39-06-4991-3340 (L.C.)
† These authors contributed equally.

Citation: Nocca, G.; D'Avenio, G.; Amalfitano, A.; Chronopoulou, L.; Mordente, A.; Palocci, C.; Grigioni, M. Controlled Release of 18-β-Glycyrrhetinic Acid from Core-Shell Nanoparticles: Effects on Cytotoxicity and Intracellular Concentration in HepG2 Cell Line. *Materials* **2021**, *14*, 3893. https://doi.org/10.3390/ma14143893

Academic Editor: Katarzyna Winnicka

Received: 11 June 2021
Accepted: 07 July 2021
Published: 12 July 2021

Publisher's Note: MDPI stays neutral with regard to jurisdictional claims in published maps and institutional affiliations.

Copyright: © 2021 by the authors. Submitted for possible open access publication under the terms and conditions of the Creative Commons Attribution (CC BY) license (http://creativecommons.org/licenses/by/4.0/).

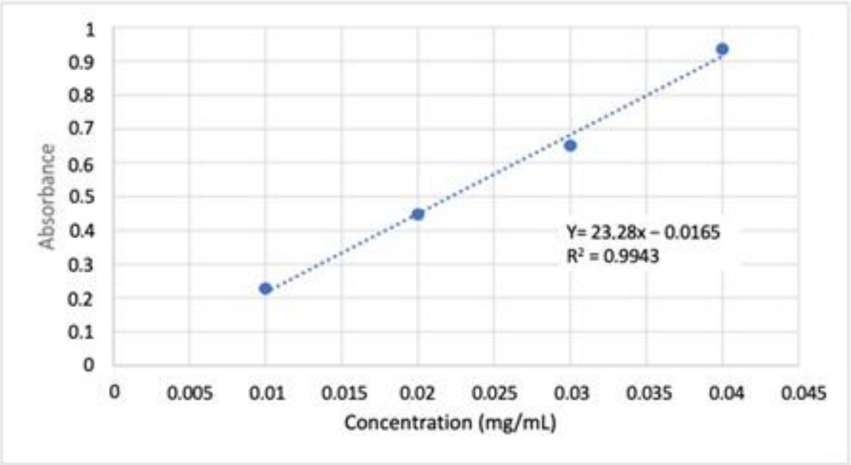

Figure S1. .GA Calibration curve in chloroform, obtained using a spectrophotometric method at $\lambda = 247$ nm.

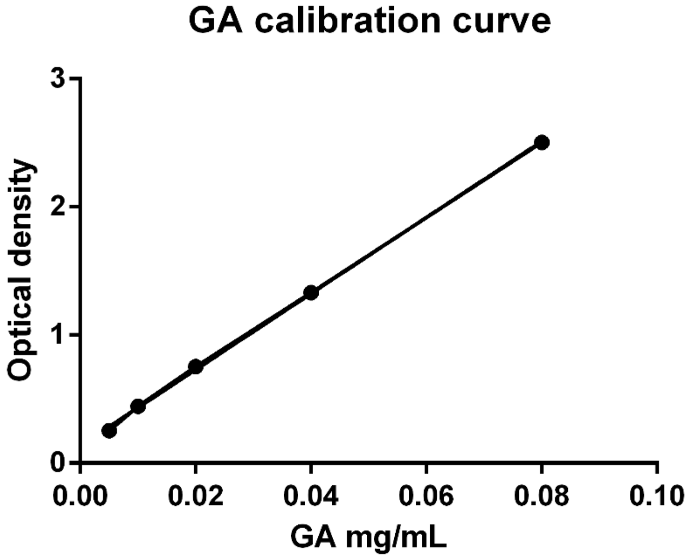

Figure S2. . GA calibration curve in PBS utilized to verify the solubility of GA.

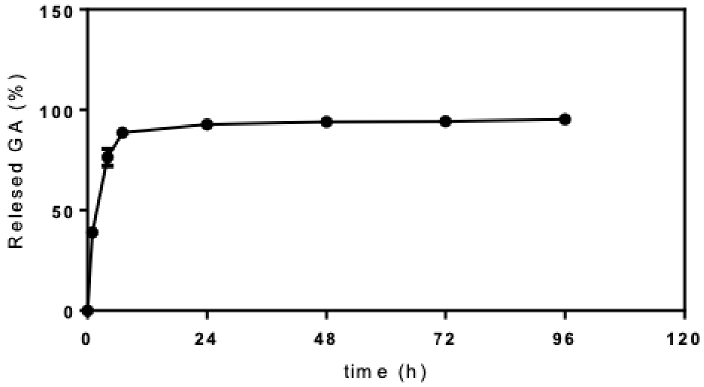

Figure S3. . GA release from GA-NPs in ethanol.
